# Peak Oxygen Uptake on Cardiopulmonary Exercise Test Is a Predictor for Severe Arrhythmic Events during Three-Year Follow-Up in Patients with Complex Congenital Heart Disease

**DOI:** 10.3390/jcdd9070215

**Published:** 2022-07-04

**Authors:** Felix von Sanden, Svetlana Ptushkina, Julia Hock, Celina Fritz, Jürgen Hörer, Gabriele Hessling, Peter Ewert, Alfred Hager, Cordula M. Wolf

**Affiliations:** 1 Department of Congenital Heart Defects and Pediatric Cardiology, German Heart Center Munich, School of Medicine & Health, Technical University of Munich, 80636 Munich, Germany; felix.v.sanden@googlemail.com (F.v.S.); ptushkina.s@googlemail.com (S.P.); hock@dhm.mhn.de (J.H.); celina.fritz88@googlemail.com (C.F.); hessling@dhm.mhn.de (G.H.); ewert@dhm.mhn.de (P.E.); hager@dhm.mhn.de (A.H.); 2 Department of Congenital and Pediatric Heart Surgery, German Heart Center of Munich, School of Medicine & Health, Technical University of Munich, 80636 Munich, Germany; hoerer@dhm.mhn.de; 3 Division of Congenital and Pediatric Heart Surgery, University Hospital, Ludwig-Maximilians-University, 81377 Munich, Germany; 4 DZHK (German Centre for Cardiovascular Research), Partner Site Munich Heart Alliance, 80802 Munich, Germany

**Keywords:** congenital heart disease (CHD), sudden cardiac death, ventricular arrhythmia, implantable automatic cardioverter defibrillator, exercise testing in congenital heart disease, adult congenital heart disease

## Abstract

Patients with congenital heart disease (CHD) are at increased risk for severe arrhythmia and sudden cardiac death (SCD). Although implantable cardioverter defibrillators (ICD) effectively prevent SCD, risk stratification for primary prophylaxis in patients with CHD remains challenging. Patients with complex CHD undergoing CPET were included in this single-center study. Univariable and backwards stepwise multivariable logistic regression models were used to identify variables associated with the endpoint of severe arrhythmic event during three years of follow-up. Cut-off values were established with receiver operating characteristic (ROC) curve analysis. Survival analysis was conducted via Kaplan–Meier plots. Severe Arrhythmia was documented in 97 of 1194 patients (8.1%/3 years). Independent risk factors for severe arrhythmia during follow-up were old age and a low peak oxygen uptake ($\dot{V}$O_2_peak) on multivariable analysis. Patients with more advanced age and with $\dot{V}$O_2_peak values of less than 24.9 mL/min/kg were at significantly increased risk for the occurrence of severe arrhythmias during follow-up. The combined analysis of both risk factors yielded an additional benefit for risk assessment. Age at CPET and $\dot{V}$O_2_peak predict the risk for severe arrhythmic events and should be considered for risk stratification of SCD in patients with complex CHD.

## 1. Introduction

Patients with congenital heart disease (CHD) face an increased long-term risk for severe arrhythmia and sudden cardiac death (SCD); up to 26% of deaths within this population are caused by SCD [1,2,3,4]. Implantable cardioverter defibrillators (ICD) are designed to treat sudden ventricular tachyarrhythmia, which is the leading cause of SCD, occurring in up to 80% of CHD patients [1,5]. Primary ICD prophylaxis is well-accepted in the adult population [6]. The SCD-HeFT [7], MADIT-II [8,9], and DEFINITE [10] trials evaluated the effects of primary ICD therapy on mortality in ischemic and non-ischemic heart failure patients and concluded that both all-cause mortality and the SCD risk were reduced in the ICD groups of the studies.

However, defibrillators may cause complications, including inappropriate shocks, which are especially frequent in children [11,12,13]. Several studies have stated that both appropriate and inappropriate ICD shocks were associated with higher mortality and reduced quality of life [14,15]. As CHD patients face an additionally high risk for ICD complications [16,17], it becomes clear that individualized risk assessment and appropriate therapy is crucial. Recent studies by Vehmeijer and colleagues [3] evinced the shortcomings of the 2015 guidelines for primary ICD implantation in CHD patients and concluded that both Consensus Statement [18] and the European Society of Cardiology (ESC) guidelines [6] yielded poor discriminative abilities for adequate ICD-implantation recommendations. As the recently published 2020 ESC Guidelines for the management of adult CHD proposed only few changes for primary ICD prophylaxis in CHD patients [19], new means for risk assessment should be evaluated.

Cardiopulmonary exercise testing (CPET) is a well-established and safe method to assess cardiopulmonary function in children and adults with CHD [20,21,22]. Despite its comprehensive testing capabilities, CPET is not mentioned as a tool for SCD risk stratification in both 2015 and 2020 guidelines [6,19].

This study aims to evaluate measurements obtained during CPET as appropriate tools of SCD-risk assessment in patients with complex CHD via the analysis of severe arrhythmia during a three-year follow-up.

## 2. Methods

### 2.1. Study Design

This study was designed as a single-center retrospective analysis of patients with complex CHD who underwent CPET at the German Heart Centre of Munich between January 2009 and December 2014. The purpose of this study was to evaluate measurements obtained during CPET as predictors for the occurrence of severe arrhythmias in a follow-up time of three years. If multiple CPETs occurred, the most recent one with a follow-up of 3 years was used. Only patients with univentricular heart (UVH), Ebstein’s anomaly (EBS), tetralogy of Fallot (TOF), truncus arteriosus communis (TAC), and transposition of the great arteries (TGA) who underwent arterial switch operation (TGA ASO) or Senning/Mustard procedure (TGA SM) were included. TGA patients with other surgical reconstructions were excluded from analysis.

### 2.2. Cardiopulmonary Exercise Test

Patients underwent an exhausting (respiratory exchange ratio >1.0) and symptom-limited CPET in an upright position on a bicycle. A protocol with a customized ramp-wise increase in workload was used, aiming for an exercise time of about 8–12 min after an unloaded three-minute warm-up and followed by a 2–3 min cool-down at 5–20 watts. Usually, we used a 10, 15, or 20 Watt/min increase in workload in patients with complex congenital heart defects. The highest running mean of any thirty-second interval of oxygen uptake during exercise was defined as peak oxygen uptake ($\dot{V}$O2peak). $\dot{V}$O2peak was expressed relative to body mass (mL/min/kg) rather than as a percentage of predicted value since the differences in cardiopulmonary anatomy and physiology between patients with complex CHD and the physiological collective used for normation were considered a potential bias. Estimation of ventilatory efficiency ($\dot{V}$E/$\dot{V}$CO2_-_slope) was defined by manually excluding the values after the respiratory compensation point [23,24].

### 2.3. Data Collection

The following data were reviewed and collected on the date of CPET: demographic and clinical data (age, gender, and body mass index (BMI)), $\dot{V}$O2peak, anaerobic threshold ($\dot{V}$O2at), $\dot{V}$E/$\dot{V}$CO2-slope, respiratory exchange ratio at peak exercise (RERmax), and pulse oxymetric saturation at peak exercise (SpO_2_max). Medical charts and available Holter recordings as well as ICD-, pacemaker- and event-recorder readings were reviewed within a follow-up time of three years after CPET. The function of the systemic ventricle in transthoracic ultrasound assessed by visual estimation or via ejection fraction measurement was added to the analysis if the examination occurred within 12 months of the initial CPET. An ejection fraction of less than 50% was considered as an impaired function. The primary endpoint was survival without severe arrhythmic events (SAE), namely sudden cardiac death (SCD), aborted SCD, appropriate implantable cardioverter defibrillator (ICD) discharge, ICD antitachycardia pacing (ICD-ATP) for ventricular tachycardia (VT), hospital admission for acute ventricular arrhythmia, cardiac syncope caused by ventricular arrhythmia, and the occurrence of non-sustained or sustained VT (nsVT, sVT) on Holter, event-recorder, pacemaker, or ICD recordings. Heart transplantation was considered as death of the patient’s heart and thus terminated follow-up. VT was defined in contrast to the ACC/AHA/HRS 2006 key data elements and definitions for electrophysiological studies and procedures [25] as wide complex tachycardia without atrial origin, exceeding three beats in succession. VTs were characterized as non-sustained if they terminated in <30 s and sustained if they persisted ≥30 s. Hospital admissions without evidence of acute VT and syncopes without complete loss of consciousness or with another cause more likely than VT were not considered as SAE.

### 2.4. Statistical Analysis

Data analyses were performed using SPSS (version 25.0, IBM Corporation, Armonk, NY, USA). Categorical variables were expressed as absolute (n/N) and relative frequencies (%). Continuous variables were expressed as means ± standard deviation (SD) or medians and interquartile range (IQR), depending on the distribution. The normality of distribution was assessed by visual analysis of plotted histograms. Homogeneity of variances was determined using Levene’s test. ANOVA (A), Kruskal–Wallis (KW), and Pearson’s chi-squared (χ^2^) tests were used to unveil statistically significant differences of values between the subgroups of CHD. If not otherwise defined, all comparisons refer to the average of the included subgroups. To account for multiple testing, the Bonferroni correction was applied.

Univariable and backwards stepwise multivariable logistic regression models were used to identify parameters associated with SAE within three years. Variables with *p*-values less than 0.10 in univariable analyses were considered in the multivariable analysis (entry-threshold, *p* = 0.05; removal-threshold *p* = 0.10). Additionally, testing for multicollinearity was conducted to determine the degree of correlation between variables. If two variables had a Pearson or Spearman-Rho correlation coefficient (PCC, SRCC) exceeding 0.70, the one with higher *p*-value in univariable analysis was excluded from the multivariable model. Results were reported as odds ratios (ORs) with 95% confidence intervals (CIs). A priori power analysis was conducted with G*Power (Version 3.1.9.7) [26]. We estimated an overall 3-year risk for SAE of 10% and a relative risk modification of 25% per SD. The necessary sample size accounting for an α-error of 0.05 and a power of 0.8 was calculated as 1118 patients. Time-to-event analysis was conducted via univariabe and backwards stepwise multivariable Cox regression analysis. Inclusion and exclusion criteria did not differ from the aforementioned logistic regression analysis and the results were reported as hazard ratios (HR) with 95% CI. To evaluate the diagnostic ability of $\dot{V}$O_2_peak, receiver operating characteristic (ROC) analysis was used, and the results were reported as areas under the curves (AUC) with 95% CI. ROC analysis was further used to find cut-off values wherever possible by determining the maximum Youden’s index (YI). The differences in SAE-free survival were visualized by the means of Kaplan–Meier estimator with time 0 as the date of CPET and log-rank testing was used to ensure statistical significance.

*p*-values < 0.05 were considered significant for all tests.

## 3. Results

### 3.1. Patient Characteristics

Patient characteristics, CPET variables, and the applied means of rhythm recording are shown in Table 1. In total, 1194 patients (663 male) were included in the study. The median age at the CPET performance was 25.9 (IQR 17.4–34.6) years, the median body height was 168 (IQR 160–175) cm, the mean body mass was 64.7 ± 18.6 kg, and the median BMI was 22.4 (IQR 19.8–25.6) kg/m^2^. The underlying diagnosis was UVH in 205 patients, EBS in 135 patients, TOF in 469 patients, TAC in 51 patients, TGA ASO in 149 patients, and TGA SM in 185 patients. Forty-nine patients with TGA underwent various different surgical reconstructions and were excluded from the analysis. Transthoracic ultrasound evaluation of systemic ventricle function was available in 1148 patients within 12 months of the initial CPET. Peak performance was reached by 1075 patients during CPET. In 66 patients, oxygen saturation could not be measured at peak of exercise. In 41 patients, the $\dot{V}$O_2_at could not be determined reliably. In both cases, the variables were omitted from the statistical analysis. Three years of follow-up were completed in 1101 patients (92.2%). Holter recordings were available in 445 patients (38.1%). During follow-up, ICDs were present in 42 patients, 145 patients had a pacemaker, and event-recorders were used in three patients, out of which 27 pacemakers and 17 ICDs were implanted during follow-up with five being ICD-upgrades to preexisting pacemakers.

Twenty-seven patients (2.3%) died during follow-up. SCD was diagnosed in two patients. Sixteen patients died from causes other than SCD. In nine patients, the cause of death remained unclear. Heart transplantation was performed in three patients. There were differences in some of the CPET measurements between the different CHD groups when compared to the total group (Table 1). However, occurrence of SAE did not differ between the distinct anatomic groups (Table 2), and the entire group was considered for further univariable and multivariable analysis.

### 3.2. Severe Arrhythmic Events during Follow-Up

Severe arrhythmic events during three-year follow-up occurred in 97 of 1194 patients (8.1%). No statistically significant differences were found among the subtypes of CHD, regarding the occurrence of SAE (Table 2). The most frequent SAE was nsVT documented by Holter-ECG, pacemaker, ICD, or event recorder (7.0%). The longest nsVTs of individual patients terminated after an average of 21.63 ± 29.69 beats, and TGA ASO patients presented with significantly shorter nsVT compared to the total average (TGA ASO 6.14 ± 2.12 vs. total 21.63 ± 29.69 beats; *p* < 0.001). Sustained VT was documented in eight patients. SCD occurred twice during follow-up, once in the EBS and once in the TOF subgroup. In six patients, SCD was aborted by the means of cardiopulmonary resuscitation (CPR). ICD intervention occurred in ten patients (ICD-ATP *n* = 8; appropriate ICD-discharge n = 6) during follow-up. Eleven patients were hospitalized due to acute VT, and 14 experienced a sudden syncope with strong suspicion of VT. In nine patients, nsVT was documented during CPET, one of which experienced an aborted sudden cardiac death with successive ICD-implantation during follow-up, and, in two patients, recurrent nsVT was identified in holter recordings. None of the examined patients had sustained VT during CPET.

### 3.3. Clinical Parameters Associated with SAE

Univariable and multivariable logistic regression analyses are summarized in Table 3. Clinical variables associated with SAE in multivariable analysis were age at CPET (OR, 1.029; 95% CI, 1.009–1.049; *p* = 0.004) and $\dot{V}$O_2_peak (OR, 0.951; 95% CI, 0.921–0.982; *p* = 0.002). $\dot{V}$O_2_ at correlated with $\dot{V}$O_2_peak (PCC, 0.83; *p* < 0.001; SRCC, 0.83; *p* < 0.001) and was excluded from multivariable analysis. BMI did correlate with SAE in univariable analysis but did not alter the risk of SAE in multivariable analysis. $\dot{V}$E/$\dot{V}$CO_2_-slope, RERmax, SpO_2_max, gender, and type of CHD did not correlate significantly with SAE during follow-up in our logistic regression model. Similar results were found in univariable and multivariable Cox regression analyses, which are depicted in Table S1.

### 3.4. The Predictive Value of $\dot{V}$O_2_peak and Age

ROC curve analysis illustrated that $\dot{V}$O_2_peak (AUC, 0.687; 95% CI, 0.631–0.743; *p*-value < 0.001) and age (AUC, 0.659; 95% CI, 0.602–0.715; *p* < 0.001) independently predicted risk for SAE in CHD patients (Figures S1 and S2). ROC analysis revealed potential cut-off values for $\dot{V}$O_2_peak at 24.9 mL/min/kg (YI, 0.318; sensitivity 0.702; specificity 0.616) and for age at 26.2 years (YI, 0.242; sensitivity 0.711; specificity 0.531); however, age especially correlated fairly linearly with the SAE risk.

### 3.5. Prediction of SAE-Free Survivial with $\dot{V}$O_2_peak and Age

**Figure 1:**
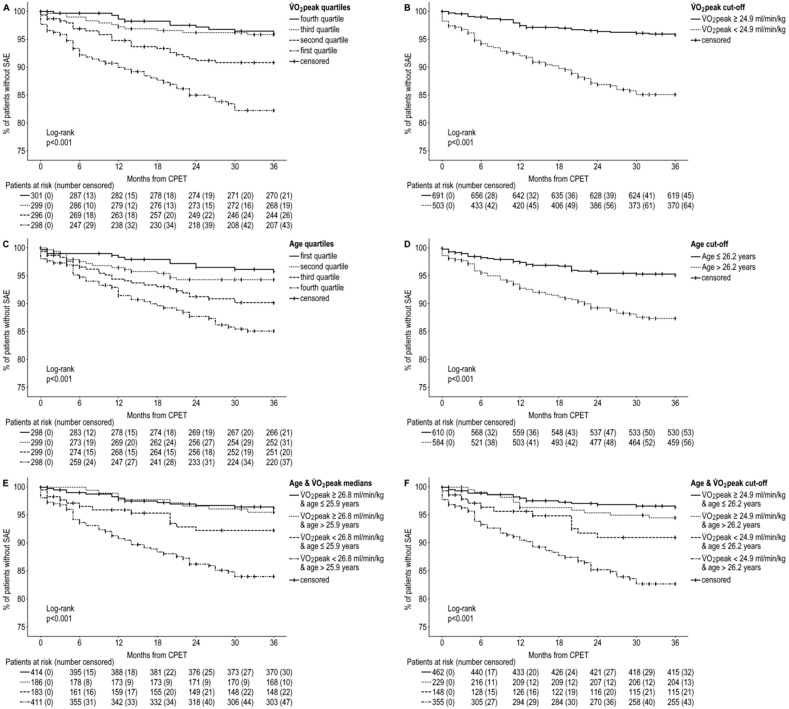
Kaplan–Meier SAE-free survival for $\dot{V}$O_2_peak and age. (**A**) Kaplan–Meier SAE-free survival for quartiles of $\dot{V}$O_2_peak, (**B**) Kaplan–Meier SAE-free survival for patients reaching or failing the ROC cut-off value for $\dot{V}$O_2_peak (24.9 mL/min/kg), (**C**) Kaplan–Meier SAE-free survival for quartiles of age at CPET, (**D**) Kaplan–Meier SAE-free survival for patients exceeding or not exceeding the ROC cut-off value for age (26.2 years) at CPET, (**E**) Kaplan–Meier SAE-free survival separated by medians of age and $\dot{V}$O_2_peak, (**F**) Kaplan–Meier SAE-free survival separated by ROC cut-off values for age at CPET (26.2 years) and $\dot{V}$O_2_peak (24.9 mL/min/kg); SAE: Severe arrhythmic event, $\dot{V}$O_2_peak: Peak oxygen uptake, ROC: Receiver operating characteristic, CPET: Cardiopulmonary exercise testing.

While survival without SAE at three years of follow-up was similar in patients with $\dot{V}$O_2_peak in the third and fourth quartile (95.8% vs. 96.1%), lower $\dot{V}$O_2_peak values in the second and first quartile were associated with a decrease in SAE-free survival (90.7% and 82.2%) (Figure 1A). Patients with $\dot{V}$O_2_peak equal to or higher than the cut-off value of 24.9 mL/min/kg presented with more frequent 3-year survival without SAE than patients with lower $\dot{V}$O_2_peak than cut-off (95.8% vs. 85.1%) (Figure 1B). Higher age correlated with an decreased survival without SAE throughout all quartiles (95.8% vs. 94.3% vs. 90.2% vs. 85.1%) (Figure 1C), and age above the cut-off value of 26.2 years lead to less frequent 3-year survival without SAE (87.4% vs. 95.1%) (Figure 1D). The combination of lower $\dot{V}$O_2_peak values on CPET and higher age increased the risk for SAEs during follow-up even more (Figure 1E,F). It was shown that older patients with below-average $\dot{V}$O2peak presented with comparably high risk for SAE during follow-up as patients among the lowest quartile of $\dot{V}$O2peak without the consideration of age.

## 4. Discussion

Our retrospective analysis identified increased age and low $\dot{V}$O_2_peak as independent risk factors for severe arrhythmic events during three-year follow-up in a wide range of complex CHD. Age tended to correlate linearly with the occurrence of SAE. We found that $\dot{V}$O_2_peak correlated non-linearly, and a cut-off value for $\dot{V}$O_2_peak of 24.9 mL/min/kg presented to be an effective margin for risk assessment. Combined risk stratification of age and $\dot{V}$O_2_peak appeared to surpass the predictive capability of isolated contemplation. There were no statistical differences between the occurrences of SAEs when comparing the distinct anatomical groups. Statistical power was limited in this comparison given the small sample sizes of patients with SAEs when breaking down to the individual anatomical groups. Extrapolation of the findings assessing SAE risk factors in a univariate and multivariate analysis in the entire heterogeneous group consisting of distinct complex structural heart diseases might not allow extrapolation to the respective anatomical subgroups. Despite these limitations, reduced $\dot{V}$O_2_peak and more advanced age were shown to be capable predictors for severe arrhythmia among a collective of various complex CHD and thus should be considered for SCD risk stratification.

In our analysis, 8.1% of patients with complex CHD experienced severe arrhythmia within three years of follow-up. No significant differences were found between the different groups of CHD, but strong trends suggest a prominent risk for severe arrhythmia in patients with Ebstein’s anomaly, while the risk in patients with a common arterial trunk appeared to be negligible. Recent studies suggest that EBS patients are at significant risk for arrhythmias and sudden cardiac death [27,28,29]. It has been stated that these lethal arrhythmias in Ebstein’s anomaly are caused by the anatomic proximity of the structural abnormalities to the conduction system, namely the tricuspid annulus, the central fibrous body, the atrioventricular (AV) node, the right-sided myocardium, and the papillary muscles [30]. The predisposition for the development of accessory atrioventricular pathways may lead to rapid conduction of atrial fibrillation or flutter, which has been described to degenerate into fatal ventricular tachycardia or fibrillation in a susceptible ventricle [30].

Despite the expected high risk for SCD in patients with complex CHD [31], SCD was the least common endpoint in our analysis, while documented ventricular tachycardia represented the majority of SAE and ICD intervention occurred relatively frequent. The high rate of ICD interventions and the low rate of SCD support the effectiveness of primary and secondary prophylaxis with ICD [17]. Compared with the other types of CHD, the TOF group presented with the highest percentage of ICD. This might indicate a more liberal approach to ICD implantation in this CHD, due to the specific recommendations in ESC guidelines concerning primary ICD prophylaxis in patients with TOF [6,19].

In CPET, $\dot{V}$O_2_peak is used as a means to measure cardiopulmonary function capacity and, in particular, to assess the severity of heart failure [32,33]. Heart failure is a leading cause for VT and SCD [34,35] in acquired heart diseases; so, the correlation between $\dot{V}$O_2_peak and severe arrhythmia appears consistent. Previous studies in congenital heart diseases have linked decreased $\dot{V}$O_2_peak and increased $\dot{V}$E/$\dot{V}$CO_2_ slope during CPET with an increased rate of mortality or ventricular tachycardia in patients with tetralogy of Fallot [36,37]. In our study, $\dot{V}$E/$\dot{V}$CO_2_ slope did not show a clear correlation with the risk of severe arrhythmia during follow-up. This may be due to the wider inclusion of CHD patients as well as the strong emphasis towards severe arrhythmia rather than mortality. The correlation of increased age and the risk for SCD is well-known in patients with CHD [38]. Still, the additional benefit of the combined interpretation of age and $\dot{V}$O_2_peak has not yet been established in the context of SCD-risk stratification.

The findings of the current study are relevant since the assessment of risk in CHD patients is still a subject of debate, with studies suggesting that the 2015 guidelines only yield poor discriminative ability for patients at risk of SCD [3,39]. Data concerning the risk stratification in patients with systemic right ventricle and univentricular heart physiology are considered especially scarce [40]. The 2020 ESC guidelines proposed only minor changes to primary ICD prophylaxis and did not mention CPET as a means for risk stratification [19]. Furthermore, specific guidelines for primary ICD prophylaxis in CHD patients remain elusive [18,41]. The analysis of $\dot{V}$O_2_peak in conjunction with the patient’s age might yield an additional means for risk stratification of severe arrhythmia and sudden cardiac death in patients with complex CHD and thus help to distinguish patients who could benefit from primary ICD prophylaxis.

### Study Limitations

This study was retrospective in nature, and, thus, general limitations for this study design apply. Although the patient cohort had been large, the examined patient group was quite heterogeneous with regard to the underlying structural heart disease. In addition, there may have been important within-group heterogeneity with regard to anatomy, type of repair, age at repair, potential reoperations, and potential residual or newly acquired defects. Also, the frequency of Holter recordings and device implantation was not standardized for this study. A selection bias favorable to a patient cohort with more complex disease could occur, as the study was carried out in a highly specialized tertiary care center.

## 5. Conclusions

With the limitations of a retrospective study design and a heterogeneous patient population, more advanced age and low $\dot{V}$O_2_peak on CPET presented as risk factors for an increased risk of severe arrhythmic events during three-year follow-up in patients with complex CHD. Age and $\dot{V}$O_2_peak on CPET should be considered for SCD risk stratification and the individualized decision for primary prophylactic ICD implantation or liberal ablation therapy if appropriate.

## Figures and Tables

**Table 1:** Patient characteristics, results of CPET, and the applied means of rhythm recording (total and separated in CHD groups).

	Total	UVH	EBS	TOF	TAC	TGA ASO	TGA SM	*p*-Value
	663/1194 (55.5)	118/205 (57.6)	60/135 (44.4)	235/469 (50.1)	31/51(60.8)	105/148 (70.3)	114/186 (61.8)	<0.001 [χ^2^]
Age [median(IQR)]	25.9 (17.4–34.6)	22.7 (13.1–30.8)	37.1 (24.9–48.2)	26.2 (18.5–35.9)	23.5 (16.1–28.4)	16.2 (13.3–19.4)	31.0 (27.1–36.5)	<0.001 [KW]
BMI in kg/m^2^ [median(IQR)]	22.4 (19.8–25.6)	21.2 (17.9–24.4)	24.1 (20.9–26.9)	22.79 (20.0–25.6)	22.2 (19.9–25.1)	20.7 (17.9–22.3)	24.2 (21.9–27.1)	<0.001 [KW]
CPET								
Peak performance reached [n/N(%)]	1075/1194 (90.0)	184/205 (89.8)	121/135 (89.6)	428/469 (91.3)	44/51(86.3)	132/148 (89.2)	166/186 (89.2)	0.866 [χ^2^]
$\dot{V}$O_2_peak in mL/min/kg [median(IQR)]	26.8 (21.3–33.3)	25.7 (19.8–32.2)	23.1 (18.4–28.9)	26.9 (21.5–33.2)	28.0 (23.4–33.5)	37.2 (29.4–43.5)	24.6 (20.5–29.4)	<0.001 [KW]
$\dot{V}$O2at in mL/min/kg [median(IQR)]	16.0 (12.4–20.1)	15.7 (11.8–20.4)	13.3 (10.7–16.8)	16.2 (12.5–19.9)	17.1 (12.8–20.3)	20.3 (17.0–25.0)	14.5 (12.1–17.2)	<0.001 [KW]
$\dot{V}$E/$\dot{V}$CO_2_-slope [median(IQR)]	28.1 (25.4–31.5)	31.9 (29.1–35.0)	28.8 (25.7–33.1)	26.8 (24.3–29.7)	27.4 (25.0–29.9)	26.5 (24.5–28.8)	29.1 (26.2–32.1)	<0.001 [KW]
RERmax [mean±SD]	1.1 ± 0.1	1.1 ± 0.1	1.2 ± 0.1	1.1 ± 0.1	1.1 ± 0.1	1.1 ± 0.1	1.1 ± 0.1	0.001 [A]
SpO_2_max in % [median(IQR)]	94.0 (91.0–97.0)	89.0 (84.0–92.0)	97.0 (93.8–98.0)	95.5 (93.0–98.0)	96.0 (93.0–97.0)	96.0 (94.0–98.0)	93.0 (90.0–95.0)	<0.001 [KW]
Impaired systemic ventricle function [n/N(%)]	160/1148 (13.9)	53/194 (27.3)	5/119 (4.2)	23/456 (5.0)	4/50 (8.0)	5/146 (3.4)	70/183 (38.3)	<0.001 [χ^2^]
Follow-up complete [n/N(%)]	1101/1194 (92.2)	198/205 (96.6)	117/135 86.7)	419/469 (89.3)	51/51 (100.0)	139/148 (93.9)	177/186 (95.2)	<0.001 [χ^2^]
Holter recordings [n/N(%)]	445/1194 (38.1)	107/205 (52.2)	58/135 (43.0)	157/469 (33.5)	17/51(33.3)	29/148 (19.6)	87/186 (46.8)	<0.001 [χ^2^]
Implanted device [n/N(%)]	175/1194 (14.7)	52/205 (25.4)	26/135 (19.3)	51/469 (10.9)	2/51(3.9)	7/148(4.7)	37/186 (19.9)	<0.001 [χ^2^]
Pacemaker [n/N(%)]	130/1194 (10.9)	49/205 (23.5)	24/135 (17.8)	21/469 (4.5)	1/51(2.0)	4/148(2.7)	31/186 (16.7)	<0.001 [χ^2^]
ICD [n/N(%)]	26/1194 (2.2)	2/205(1.0)	2/135(1.5)	17/469(3.6)	0/51(0.0)	1/148(0.7)	4/186(2.2)	0.109 [χ^2^]
ICD & pacemaker [n/N(%)]	16/1194 (1.3)	1/205(0.5)	0/135(0.0)	11/469(2.3)	0/51(0.0)	2/148(1.4)	2/186(1.1)	0.197 [χ^2^]
Event recorder [n/N(%)]	3/1194(0.0)	0/205(0.0)	0/135(0.0)	2/469(0.4)	1/51(2.0)	0/148(0.0)	0/186(0.0)	0.145 [χ^2^]
Death during follow-up [n/N(%)]	27/1194 (2.3)	8/205(3.9)	9/135(6.7)	8/469(1.7)	1/51(2.0)	0/148(0.0)	1/186(0.5)	<0.001 [χ^2^]
Heart transplantation [n/N(%)]	3/1194(0.3)	1/205(0.5)	1/135(0.7)	0/469(0.0)	0/51(0.0)	0/148(0.0)	1/186(0.5)	0.544 [χ^2^]

CHD: Congenital heart disease, UVH: Univentricular heart, EBS: Ebstein’s disease, TOF: Tetralogy of Fallot, TAC: Truncus arteriosus communis, TGA: Transposition of the great arteries, ASO: arterial switch operation, SM: Senning/Mustard, n/N(%): Absolute and relative frequency, IQR: Interquartile range, SD: Standard deviation, BMI: Body mass index, CPET: Cardiopulmonary exercise testing, $\dot{V}$O_2_peak: Peak oxygen uptake, $\dot{V}$O_2_ at: Oxygen uptake at anaerobic threshold, $\dot{V}$E/$\dot{V}$CO_2_-slope: Estimated ventilatory efficiency, RERmax: Respiratory exchange ratio at peak exercise, SpO_2_max: Peripheral oxygen saturation at peak exercise, ICD: Implantable cardioverter defibrillator, χ^2^: Pearson’s chi-squared, KW: Kruskal-Wallis, A: ANOVA, CHD: Congenital heart disease. Echocardiographic measurement of systemic ventricle function was available in 1148 Patients.

**Table 2:** Occurrence of severe arrhythmic events (total and separated in CHD groups).

[n/N[%)]	Total	UVH	EBS	TOF	TAC	TGA ASO	TGA SM	*p*-Value
Severe arrhythmic event	97/1194(8.1)	18/205(8.8)	15/135(11.1)	41/469(8.7)	1/51(2.0)	8/149(5.4)	14/185(7.6)	0.291 [χ^2^]
SCD equivalent	15/1194(1.3)	2/205(1.0)	3/135(2.2)	8/469(1.7)	0/51(0.0)	1/149(0.7)	1/185(0.5)	0.588 [χ^2^]
SCD	2/1194(0.2)	0/205(0.0)	1/135(0.7)	1/469(0.2)	0/51(0.0)	0/148(0.0)	0/186(0.0)	0.593 [χ^2^]
Aborted SCD	6/1194(0.5)	1/205(0.5)	1/135(0.7)	2/169(0.4)	0/51(0.0)	1/149(0.7)	1/185(0.5)	0.990 [χ^2^]
ICD-ATP	8/1194(0.7)	2/205(1.0)	1/135(0.7)	5/469(1.1)	0/51(0.0)	0/149(0.0)	0/185(0.0)	0.549 [χ^2^]
Appropriate ICD-discharge	6/1194(0.5)	1/205(0.5)	1/135(0.7)	4/469(0.9)	0/51(0.0)	0/149(0.0)	0/185(0.0)	0.661 [χ^2^]
Hospitalisation/Syncope	21/1194(1.8)	2/205(1.0)	2/135(1.5)	13/469(2.8)	0/51(0.0)	2/149(1.3)	2/185(1.1)	0.400 [χ^2^]
Hospitalisation	11/1194(0.9)	1/205(0.5)	1/135(0.7)	7/469(1.5)	0/51(0.0)	1/149(0.7)	1/185(0.5)	0.697 [χ^2^]
Syncope	14/1194(1.2)	1/205(0.5)	2/135(1.5)	8/469(1.7)	0/51(0.0)	1/149(0.7)	2/185(1.1)	0.695 [χ^2^]
sVT/nsVT in device	83/1194(7.0)	17/205(8.3)	12/135(8.9)	33/469(7.0)	1/51(2.0)	7/149(4.7)	13/185(7.0)	0.481 [χ^2^]
sVT in device	8/1194(0.7)	2/205(1.0)	1/135(0.7)	4/469(0.9)	0/51(0.0)	0/149(0.0)	1/185(0.5)	0.859 [χ^2^]
nsVT in device	82/1194(6.9)	17/205(8.3)	12/135(8.9)	32/469(6.8)	1/51(2.0)	7/149(4.7)	13/185(7.0)	0.475 [χ^2^]

CHD: Congenital heart disease, UVH: Univentricular heart, EBS: Ebstein’s disease, TOF: Tetralogy of Fallot, TAC: Truncus arteriosus communis, TGA: Transposition of the great arteries, ASO: Arterial switch operation, SM: Senning/Mustard, SCD: Sudden cardiac death, ICD: Implantable cardioverter defibrillator, ATP: Antitachycardia pacing, sVT: Sustained Ventricular Tachycardia, nsVT: Non-sustained ventricular Tachycardia, device: ICD/pacemaker/event recorder, n/N(%): Absolute and relative frequency, χ^2^: Pearson’s chi-squared.

**Table 3:** Clinical parameters associated with SAE in univariable and multivariable logistic regression analysis.

	Univariable Analysis			Multivariable Analysis		
Variable	OR	95% CI	*p*-value	OR	95% CI	*p*-value
Base data						
Age [per additional year]	1.046	1.030–1.063	<0.001	1.029	1.009–1.049	0.004
BMI [per 1 kg/m^2^ increase]	1.069	1.025–1.115	0.002			
Gender [female]	1.107	0.725–1.692	0.639			
CPET						
$\dot{V}$O_2_ peak [per 1 mL/min/kg decrease]	1.078	1.048–1.107	<0.001	1.052	1.018–1.086	0.002
$\dot{V}$O_2_ at [per 1 mL/min/kg decrease]	1.098	1.050–1.148	<0.001			
VE/$\dot{V}$CO_2_-slope [per 1 increase]	1.005	0.968–1.043	0.797			
RERmax [per 1 increase]	1.741	0.199–15.214	0.616			
SpO_2_max [per 1% decrease]	1.005	0.970–1.042	0.767			
CHD						
UVH	1.088	0.635–1.864	0.758			
EBS	1.685	0.935–3.035	0.083			
TOF	1.170	0.761–1.798	0.474			
TAC	0.206	0.028–1.507	0.120			
TGA ASO	0.617	0.292–1.302	0.205			
TGA SM	0.831	0.452–1.528	0.551			

SAE: Severe arrhythmic event, OR: Odds ratio, CI: Confidence interval, BMI: Body mass index, CPET: Cardiopulmonary exercise testing, $\dot{V}$O_2_peak: Oxygen uptake at peak exercise, $\dot{V}$O_2_at: Oxygen uptake at anaerobic threshold,$\dot{V}$E/$\dot{V}$CO_2_-slope: Estimated ventilatory efficiency, RERmax: Respiratory exchange ratio at peak exercise, SpO_2_max: Pulse oxymetric saturation at peak exercise, UVH: Univentricular heart, EBS: Ebstein’s disease, TOF: Tetralogy of Fallot, TAC: Truncus arteriosus communis, TGA: Transposition of the great arteries, ASO: arterial switch operation, SM: Senning/Mustard. Individual CHD subgrups were compared to a composite of the remaining CHD. Variables with a *p*-value of less than 0.10 in univariable analysis were included to the multivariable model and underwent backwards stepwise regression. $\dot{V}$O_2_at was excluded from multivariable analysis, due to correlation with $\dot{V}$O_2_peak.

## Data Availability

The data presented in this study are available on request from the corresponding author. The data are not publicly available due to preservation of patient anonymity and privacy.

## Supplementary Materials

The following supporting information can be downloaded at: https://www.mdpi.com/article/10.3390/jcdd9070215/s1, Figure S1: ROC curve for prediction of SAE via $\dot{V}$O2peak in CHD; Figure S2: ROC curve for prediction of SAE via age in CHD; Table S1: Clinical parameters associated with SAE in univariable and multivariable Cox-regression analysis.
