# Supplementary material for: Peak Oxygen Uptake on Cardiopulmonary Exercise Test Is a Predictor for Severe Arrhythmic Events during Three-Year Follow-Up in Patients with Complex Congenital Heart Disease
Source: J Cardiovasc Dev Dis. 2022 Jul 4;9(7):215. doi: 10.3390/jcdd9070215 (PMC9320423; doi:10.3390/jcdd9070215)
Supplement: Supplementary file 1 [file jcdd-09-00215-s001.zip › jcdd-1726101-supplementary.pdf]

<u>**Supplement Material**</u>

Table S1: Clinical parameters associated with SAE in univariable and multivariable Cox-regression analysis.

	Univariable analysis			Multivariable analysis		
Variable	HR	95% CI	P-value	HR	95% CI	P-value
Base data						
Age [per additional year]	1.042	1.027-1.056	<0.001	1.023	1.006-1.041	0.009
BMI [per 1kg/m^2 increase]	1.062	1.022-1.104	0.002			
Gender [female]	1.071	0.718-1.595	0.739			
CPET						
$\dot{V}O_2$peak [per 1 ml/min/kg decrease]	1.078	1.078-1.105	<0.001	1.056	1.025-1.087	<0.001
$\dot{V}O_2$at [per 1 ml/min/kg decrease]	1.101	1.056-1.148	<0.001			
$\dot{V}E/\dot{V}CO_2$-slope [per 1 increase]	1.008	0.974-1.043	0.657			
RERmax [per 1 increase]	2.147	0.285-16.202	0.459			
SpO_2max [per 1% decrease]	1.004	0.971-1.038	0.798			
CHD						
UVH	0.926	0.555-1.545	0.769			
EBS	0.642	0.370-1.114	0.115			
TOF	0.862	0.576-1.290	0.470			
TAC	4.608	0.643-33.051	0.129			
TGA ASO	1.642	0.796-3.385	0.179			
TGA SM	1.123	0.638-1.979	0.687			

SAE: Severe arrhythmic event, HR: Hazard-ratio, CI: Confidence-Interval, BMI: Body mass index, CPET: Cardiopulmonary exercise testing, $\dot{V}O_2$peak: Oxygen uptake at peak exercise, $\dot{V}O_2$at: Oxygen uptake at anaerobic threshold, $\dot{V}E/\dot{V}CO_2$-slope: Estimated ventilatory efficiency, RERmax: Respiratory exchange ratio at peak exercise, SpO_2max: Pulse oxymetric saturation at peak exercise, UVH: Univentricular heart, EBS: Ebsteins disease, TOF: Tetralogy of Fallot, TAC: Truncus arteriosus communis, TGA: Transposition of the great arteries, ASO: arterial switch operation, SM: Senning/Mustard. Individual CHD subgrups were compared to a composite of the remaining CHD. Variables with a p-value of less than 0.10 in univariable analysis were included to the multivariable model and underwent backwards stepwise regression, $\dot{V}O_2$at was excluded from multivariable analysis, due to correlation with $\dot{V}O_2$peak.

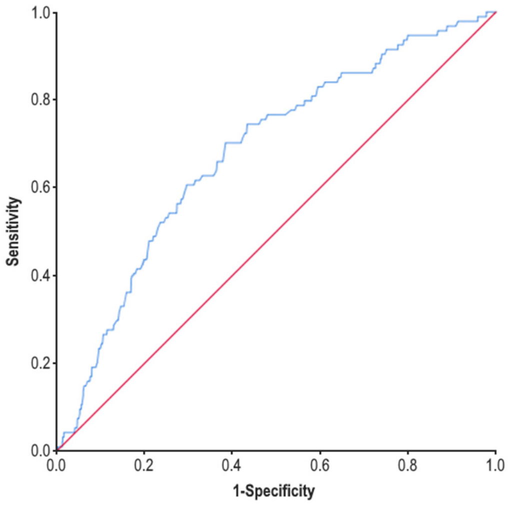

Figure S1: ROC curve for prediction of SAE via $\dot{V}O_2$peak in CHD.

ROC: receiver operating characteristic, SAE: Severe arrhythmic event, $\dot{V}O_2$peak: peak oxygen uptake, CHD: Congenital heart disease.

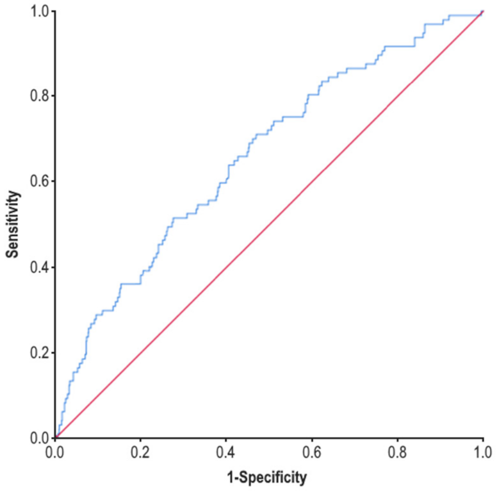

Figure S2: ROC curve for prediction of SAE via age in CHD.

ROC: receiver operating characteristic, SAE: Severe arrhythmic event, CHD: Congenital heart disease.
